# Trophodynamics of Southern Ocean pteropods on the southern Kerguelen Plateau

**DOI:** 10.1002/ece3.5380

**Published:** 2019-06-20

**Authors:** Christine K. Weldrick, Rowan Trebilco, Diana M. Davies, Kerrie M. Swadling

**Affiliations:** ^1^ Institute for Marine and Antarctic Studies University of Tasmania Hobart Tasmania Australia; ^2^ Antarctic Climate and Ecosystems Cooperative Research Centre University of Tasmania Hobart Tasmania Australia; ^3^ CSIRO Oceans and Atmosphere Hobart Tasmania Australia

**Keywords:** *Clio pyramidata*, *Clione limacina*, isotopic niche, size‐based, *Spongiobranchaea australis*, trophic position

## Abstract

Pteropods are a group of small marine gastropods that are highly sensitive to multiple stressors associated with climate change. Their trophic ecology is not well studied, with most research having focused primarily on the effects of ocean acidification on their fragile, aragonite shells. Stable isotopes analysis coupled with isotope‐based Bayesian niche metrics is useful for characterizing the trophic structure of biological assemblages. These approaches have not been implemented for pteropod assemblages. We used isotope‐based Bayesian niche metrics to investigate the trophic relationships of three co‐occurring pteropod species, with distinct feeding behaviors, sampled from the Southern Kerguelen Plateau area in the Indian Sector of the Southern Ocean—a biologically and economically important but poorly studied region. Two of these species were gymnosomes (shell‐less pteropods), which are traditionally regarded as specialist predators on other pteropods, and the third species was a thecosome (shelled pteropod), which are typically generalist omnivores. For each species, we aimed to understand (a) variability and overlap among isotopic niches; and (b) whether there was a relationship between body size and trophic position. Observed isotopic niche areas were broadest for gymnosomes, especially *Clione limacina antarctica*, whose observed isotopic niche area was wider than expected on both δ^13^C and δ^15^N value axes. We also found that trophic position significantly increased with increasing body length for *Spongiobranchaea australis*. We found no indication of a dietary shift toward increased trophic position with increasing body size for *Clio pyramidata* f. *sulcata*. Trophic positions ranged from 2.8 to 3.5, revealing an assemblage composed of both primary and secondary consumer behaviors. This study provides a comprehensive comparative analysis on trophodynamics in Southern Ocean pteropod species, and supports previous studies using gut content, fatty acid and stable isotope analyses. Combined, our results illustrate differences in intraspecific trophic behavior that may be attributed to differential feeding strategies at species level.

## 1 INTRODUCTION

Human‐driven climate change is causing significant chemical and physical changes to global oceans, leading to demonstrable direct and indirect ecological impacts to Southern Ocean assemblages across all trophic levels (Constable et al., 2014). Among these changes is ocean acidification, which causes dissolution of shell‐secreting organisms (Fabry, Seibel, Feely, & Orr, 2008; Orr et al., 2005). Acidification and warming of polar waters is also expected to lead to changing size and composition of phytoplankton and microbial communities, which could have severe bottom‐up effects on marine food webs (Davidson et al., 2016). Many shell‐secreting organisms rely on these basal food sources, and if the availability of these sources is altered, it is unclear how this will impact grazing organisms, be it through range redistribution, in situ adaptation, or localized extinction.

The pitting of aragonite shells produced by thecosome (shelled) pteropods makes them a useful biological indicator of ocean acidification due to increased atmospheric CO_2_ (Bednaršek, Harvey, Kaplan, Feely, & Možina, 2016). Thecosomes are often dominant in macrozooplankton communities within the Southern Ocean (Hunt et al., 2008), and often regarded as strict herbivores (Hopkins, 1987; Karleskint, Turner, & Small, 2012) capable of exerting significant top‐down control of phytoplankton assemblages (Perissinotto, 1992). However, gut contents have revealed the presence of metazoans such as tintinnids, copepods, and larval thecosome pteropods, indicating that they are more likely omnivorous (Hopkins & Torres, 1989). Gut content and fatty acid analyses of gymnosome (shell‐less) pteropods point to a highly specialized carnivorous diet, preying exclusively on thecosomes (Conover & Lalli, 1972). Both thecosomes and gymnosomes are important food for higher trophic level organisms (Lalli & Gilmer, 1989; Pakhomov, Perissinotto, & McQuaid, 1996), so changes to pteropod assemblages in the Southern Ocean will have flow on effects up the food web (Suprenand & Ainsworth, 2017). Despite their importance, our knowledge of pteropod trophodynamics is very patchy, particularly in the Southern Ocean, and based almost entirely on visual analysis of their stomach contents. Co‐occurring Southern Ocean pteropods could provide a unique model community to understand how different functional traits (e.g., feeding structures, body size) correlate with the trophic structure of communities consisting of both monophagous specialists and generalists.

The niche concept has been popular for over 100 years as it is a useful way of considering how different organisms fit into communities and ecosystems, and the roles they play (Grinnell, 1917; Sexton, Montiel, Shay, Stephens, & Slatyer, 2017). Several different versions of the niche concept have been introduced over time that focus on different aspects of species’ ecologies including traits, trophic interactions (resource use), and environmental (habitat) requirements (Elton, 1927; Hutchinson, 1957). Over the last decade, stable isotopes have gained popularity as an approach for quantifying the trophic niche (thus being most closely analogous to the Eltonian niche, and the resource axes of the Hutchinsonian niche) (Newsome, del Rio, Bearhop, & Phillips, 2007). This approach is based on the detectable average enrichments of 3–5‰ and ~1‰ in the isotopic ratios for nitrogen and carbon, respectively, per tropic level (Post, 2002). Measuring space occupied by consumers δ^13^C and δ^15^N enables insight into both trophic and ecological niches, as it provides both an index of the basal resources from which consumers derive their energy, and the trophic position at which they feed, respectively (Fink, Reichwaldt, Harrod, & Rossberg, 2012). As for the latter, δ^15^N values of predators rarely overlap with those of their prey, and are thus effective at estimating trophic position (Vanderklift & Ponsard, 2003).

The extent of stable isotopic values representing all resources used by a consumer is commonly used as a proxy for trophic and ecological niche (Bearhop, Adams, Waldron, Fuller, & Macleod, 2004; Jackson, Inger, Parnell, & Bearhop, 2011). Bearhop et al. (2004) first inferred the qualitative description of the trophic niche breadth by using δ^13^C and δ^15^N values of consumers and food source tissues, which was followed by the development of quantitative metrics to measure niche width, species spacing, density, overlapping, and clustering from the extent and spread of isotopic data points (Jackson et al., 2011; Layman, Quattrochi, Peyer, & Allgeier, 2007; Swanson et al., 2014). It is often assumed that smaller isotopic niches are associated with trophic specialists due to a low diversity of dietary resources, that is, consequently translated to small ranges in isotopic composition (Bolnick et al., 2003).

The niche variation hypothesis (Van Valen, 1965) proposed that broader, population‐level dietary niches are associated with greater individual‐level resource use, in relation to populations possessing narrower niches. Assuming the niche variation hypothesis, our objective was to measure trophodynamic variability in sympatric pteropod species in an under‐surveyed region on the southern Kerguelen Plateau. Here, we enlisted prior knowledge of gymnosome and thecosome pteropod feeding preferences (Lalli & Gilmer, 1989) and predicted that specialist gymnosomes will exhibit narrower isotopic niche areas than generalist thecosomes, with little variability over space and time scales. Based on studies using nitrogen stable isotopes and a range of biomass size‐spectra to show positive relationships between trophic position and body size (France, Chandler, & Peters, 1998; Jennings, Pinnegar, Polunin, & Warr, 2002), we assumed that larger individuals will have higher trophic positions than smaller individuals due increasing ability to access larger, higher trophic‐level prey with increasing body size. The effects of climate change are expected to cause shifts in the trophodynamics of many polar organisms (Gutt et al., 2015), including predictions made that may see narrowing of niche areas due to decreased prey diversity, an increase in niche overlapping due to shifts in species distribution (Bas et al., 2019), and an increase in trophic positions of key marine predators due to expected diet shifts toward higher trophic prey (Tarroux, Lowther, Lydersen, & Kovacs, 2016). The trophic niches estimated here provide a benchmark for pteropods and a piece of the puzzle for ongoing efforts to understand trophic structuring of pelagic communities and ecosystems more generally.

## 2 MATERIALS AND METHODS

### 2.1 Study area and sampling

**Figure 1:**
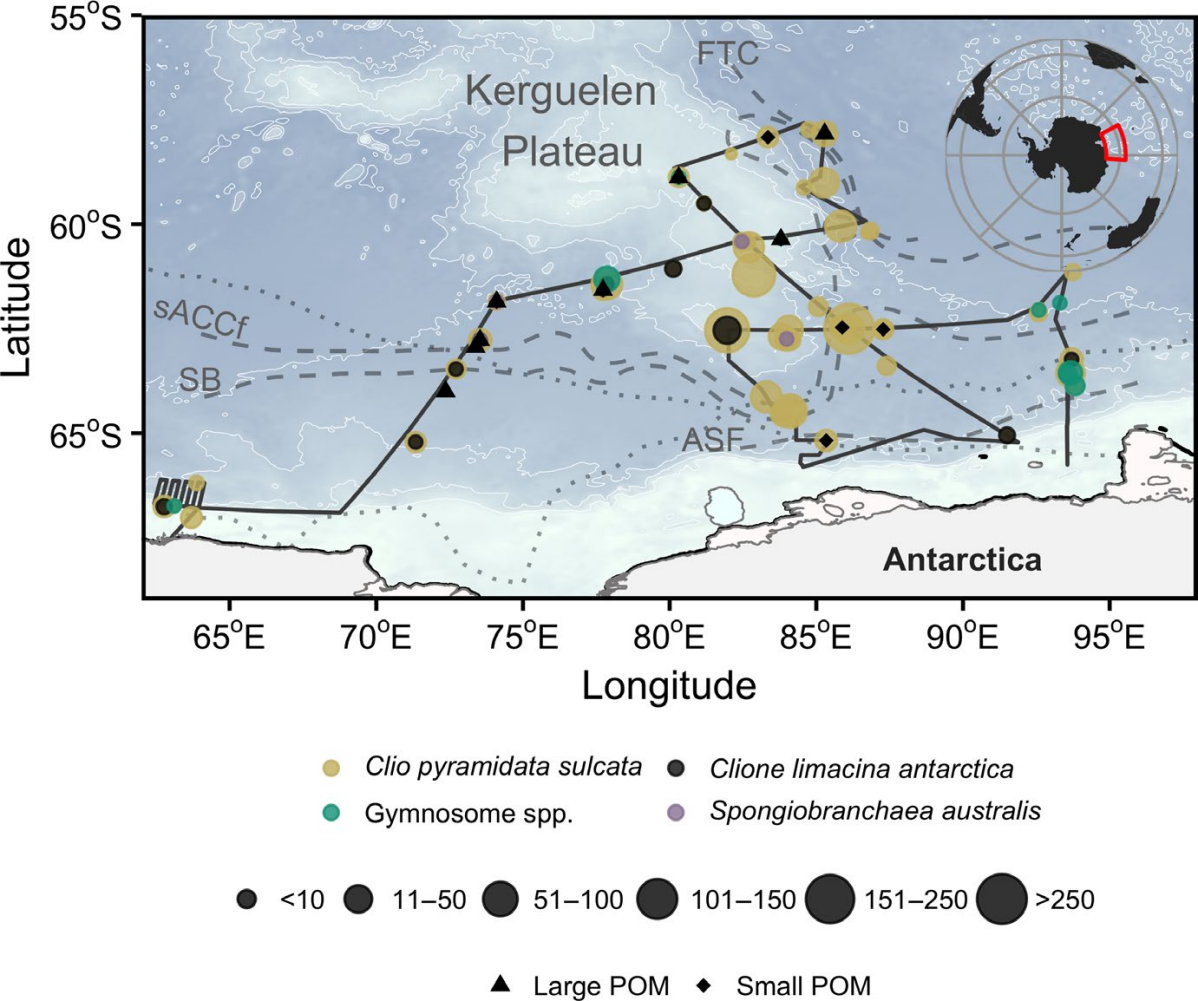
Relative abundance (size of circle, ind. 1,000 m^−3^) of pteropod species sampled from RMT8 plankton nets during the K‐Axis voyage. Some gymnosome individuals were unable to be identified to species level and were not used in SIA. Large‐fraction (triangles) and small‐fraction (diamonds) are also featured here. Oceanographic features and front locations (dashed lines) determined by Bestley et al. (2018) include the southern ACC front (sACCf), the Southern Boundary (SB), the Antarctic Slope Front, and the Fawn Trough Current. Upper and lower dotted lines are October and January average sea ice extent, respectively

Samples were collected during the Kerguelen Axis (K‐Axis) research voyage aboard the RV *Aurora Australis* between 11 January 2016 and 24 February 2016. Forty‐seven stations spanned the region from 62.7°E to 93.5°E, and from 57.6°S to 65.2°S, covering the southern extent of the Kerguelen Plateau in the Indian sector of the Southern Ocean (Figure 1). Station depths ranged from 1,276 to 4,770 m.

Macrozooplankton were sampled with a Rectangular Midwater Trawl (RMT8; mesh size: 4.5 mm, mouth area: 8 m^2^), equipped with a flow meter, and towed obliquely from the surface to 200 m. Ship speed during plankton net tows was 2–2.5 knots for an average duration of 45 min. Pteropods were counted and identified to species level, placed in cryotubes, then stored in liquid nitrogen prior to analyses. Abundances of each species were quantified in terms of individuals per 1,000 m^−3^ of seawater filtered.

Size‐fractioned particulate organic matter (POM) was retrieved from the ship's underway water supply (~5 m depth) via large volume sequential filtration, prescreened using an upstream 47 mm diameter, 1 mm filter mesh for zooplankton removal, and collected onto 25 mm diameter Sterlitech silver membrane (pore size = 1.2 µm) and Nitex filters (pore size = 210 µm). Two particle size fractions were analyzed as “large” >210 µm and “small” <210 µm, predominantly phytoplankton. The large fraction on Nitex filters was cleanly transferred to silver membranes for analysis. Filters were acidified and air dried at 60°C prior to stable isotopes analysis (SIA).

### 2.2 SIA sample preparation

Individual pteropods were rinsed in filtered seawater and weighed prior to drying in an oven for 24 hr at 60°C. While previous research recommends acidifying thecosomatous pteropods to remove carbonate material that would otherwise bias stable isotopes results (Pomerleau, Winkler, Sastri, Nelson, & Williams, 2014), the pressure from the RMT8 sampling method completely stripped entire shells from most shelled pteropods, and we did not need to acid‐treat our samples prior to further analyses as shell fragments could be easily removed using forceps. Individual dry weights were measured and samples were ground to powder using an agate mortar and pestle, then weighed into tin cups in preparation for isotopic analysis. Since lipids may bias carbon isotope ratios (Post et al., 2007), a pteropod‐specific correction offset value determined by Weldrick, Trebilco, and Swadling (2019) was used to correct bulk samples. POM sample filters were acidified dried for 48 hr at 60°C prior subsampling 5 mm diameter aliquots into silver capsules (Sercon SC0037).

### 2.3 Stable isotopes analysis

Bulk carbon and nitrogen stable isotope ratios were obtained using an automated Elementar vario PYRO cube analyser interfaced with a continuous flow IsoPrime100 isotope ratio mass spectrometer for pteropods, and a Thermo Scientific Flash 2000HT analyser coupled with a Thermo Fisher Delta V Plus mass spectrometer through a Thermo Fisher Conflo IV for POM. For pteropods, SIA was performed at the Central Science Laboratory (CSL), University of Tasmania (Sandy Bay, Tasmania), and for POM, at the Australian Nuclear Science and Technology Organisation (ANSTO, Lucas Heights, Sydney). Isotopic ratios were expressed in delta (δ) notation and reported as parts per thousand (‰) deviations from conventional certified isotopic reference standards, Vienna Pee Dee Belemnite (for carbon) and atmospheric air (for nitrogen) (DeNiro & Epstein, 1978). At CSL, laboratory working standards of sulfanilamide were repeated every 6th sample for both isotopes to measure instrument stability and precision. Average standard deviations on triplicate measurements were 0.15‰ and 0.19‰ for δ^13^C and δ^15^N values of pteropods, respectively. At ANSTO, POM isotopic data are reported relative to IAEA secondary certified standards with a standard error of analysis to 1 standard deviation (*SD*) measured at ±0.3 mil. The carbon and nitrogen percentages, by weight, were converted to atomic C:N ratios.

### 2.4 Statistical analysis

Data analyses were conducted using the statistical software R, version 3.4.0 (R Development Core Team, 2017). We used Shapiro–Wilk's and Levene's tests on isotopic values (δ^13^C and δ^15^N) to assess normality and homogeneity of variance, respectively. A multivariate analysis of variance (MANOVA; using the MANOVA function in base R) was also used to test for spatial (latitude, longitude, and site depth) and species effects (independent variables) on bulk isotopic values (dependent variables). Simple linear regressions were used to test the relationships between each isotopic values (δ^13^C and δ^15^N) and time, latitude, longitude, and site depth, as well as relationships between body size and trophic position (lm function, base R). Intraspecific comparisons of niche metrics could not be conducted by site as sample sizes of gymnosome species were lower than empirically determined recommended sizes (Syväranta, Lensu, Marjomäki, Oksanen, & Jones, 2013).

### 2.5 Niche dispersion metrics, trophic position, and body size

Interspecies isotopic niche widths were estimated using the Stable Isotope Bayesian Ellipses package in R (SIBER) version 2.1.3 (Jackson et al., 2011). SIBER calculates bivariate standard ellipses corresponding to isotopic niches, and standard ellipse areas (SEA) representing standard deviation of the data. Standard area ellipses corrected for sample sizes (SEAc) were also calculated (Jackson et al., 2011). Interspecies dietary niche area overlaps were calculated using nicheRover in R version 1.0 (Swanson et al., 2014), which uses a Bayesian framework to produce probable pairwise comparisons between the niche region of each species combination. The niche region of a species was defined at 95% and 99% probability that a species will be located within isotopic bivariate space; similarly, the niche overlap is defined at 95% and 99% probability that the niche region of one species will overlap with that of another.

We also calculated multiple probability estimates of the trophic positions of each pteropod species. Unlike the trophic level, which refers to categories of trophic modes based on integer values, the trophic position of a species is a continuous, numerical measure that represents the relative location of a particular species within a trophic hierarchy (Carscallen, Vandenberg, Lawson, Martinez, & Romanuk, 2012). Trophic positions were calculated for each pteropod species separately using the R package tRophicPosition version 0.7.5 (Quezada‐Romegialli et al., 2018). This package calculates consumer trophic positions within a Bayesian framework based on the stable isotopes of both consumers and baselines as well as user‐specified trophic enrichment factors (TEF). We adopted TEF values (±*SE*) of 0.5 ± 0.13‰ for carbon (∆δ^13^C values) and 2.3 ± 0.18‰ for nitrogen (∆δ^15^N values) based on those measured for similarly sized marine gastropods previously reported in the literature (McCutchan, Lewis, Kendall, & McGrath, 2003). We also converted δ^15^N values to trophic positions on specimens used to compare with body size. Body lengths (mm) from a subset of intact pteropods were measured using ImageJ/Fiji software (Schindelin et al., 2012). Results from individuals sampled for SIA were plotted against δ^15^N values to determine the effect of body size on trophic position.

## 3 RESULTS

### 3.1 Species abundance and composition

One thecosome (shelled) and two gymnosome (shell‐less) pteropod species were identified: *Clio pyramidata*, *Clione limacina antarctica* (hereafter referred to as *C. pyramidata* and *C. antarctica*, respectively), and *Spongiobranchaea australis*. *C. pyramidata* were the most abundant pteropod in the region, followed by the gymnosome species, with averages of 22 (maximum: 272) and 2 (maximum: 47) ind. 1,000 m^−3^, respectively. Relative abundances were 89.6% for *C. pyramidata*, 4.8% for *C. antarctica*, 0.4% for *S. australis*, and 5.2% for gymnosomes unidentified to species level that were not used in the subsequent SIA (Figure 1). Latitudinal ranges were narrowest for *S. australis* (60.4°S–62.7°S), followed by *C. antarctica* (61.4°S–67.0°S), and *C. pyramidata* (57.7°S–67.0°S).

### 3.2 Bulk isotopic values of pteropods

**Figure 2:**
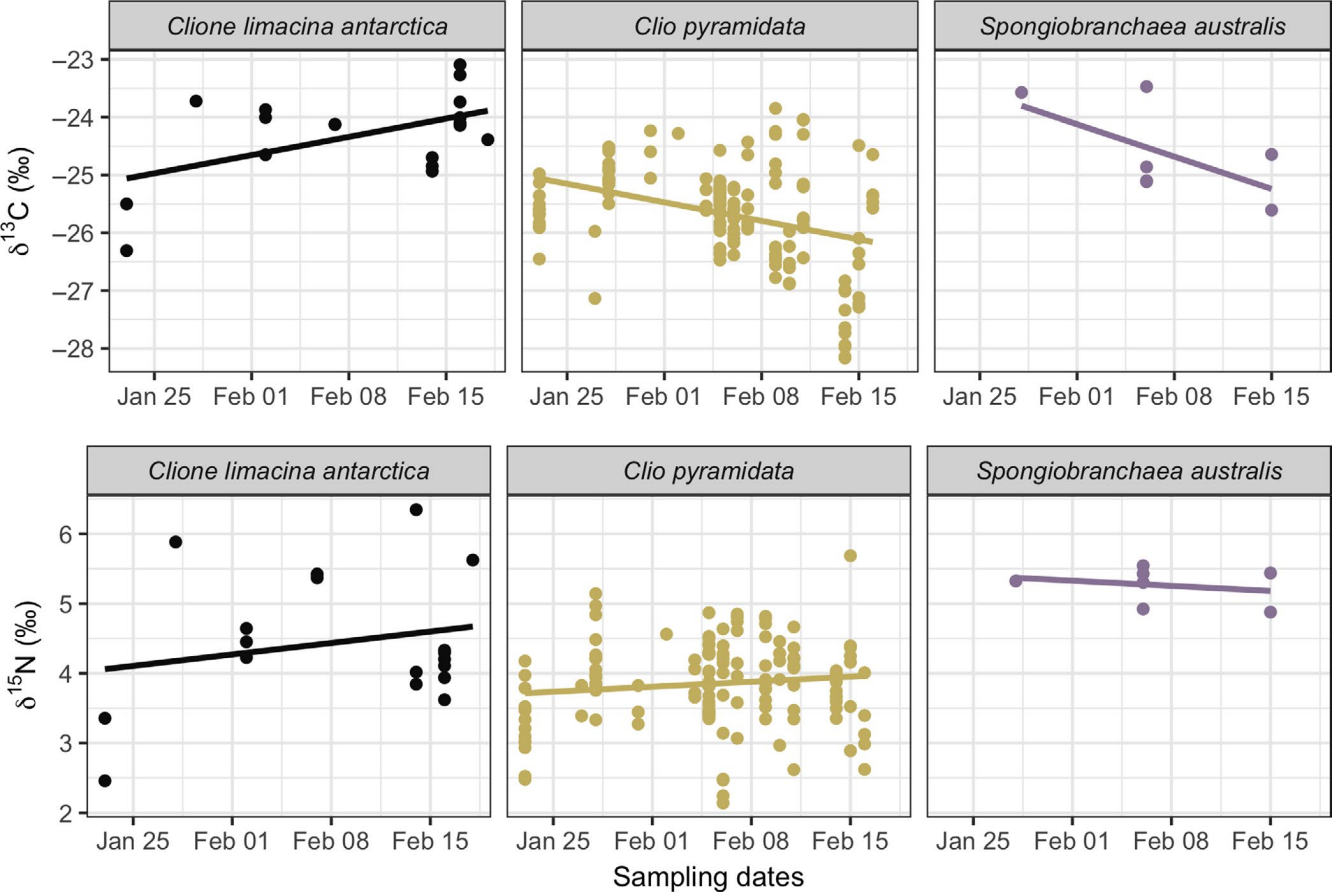
Linear relationships between δ^13^C (upper plots) and δ^15^N (lower plots) and sampling dates. For *C. antarctica*: δ^13^C, *R* ^2^ = 0.50, *p* < 0.05, δ^15^N, *R* ^2^ = 0.34, *p* < 0.05; *C. pyramidata*: δ^13^C, *R* ^2^ = 0.21, *p* < 0.05, δ^15^N, *R* ^2^ = 0.06, *p* < 0.05; *S. australis*: δ^13^C, *R* ^2^ = 0.11, *p* = 0.35, δ^15^N, *R* ^2^ = −0.39, *p* = 0.85

**Figure 3:**
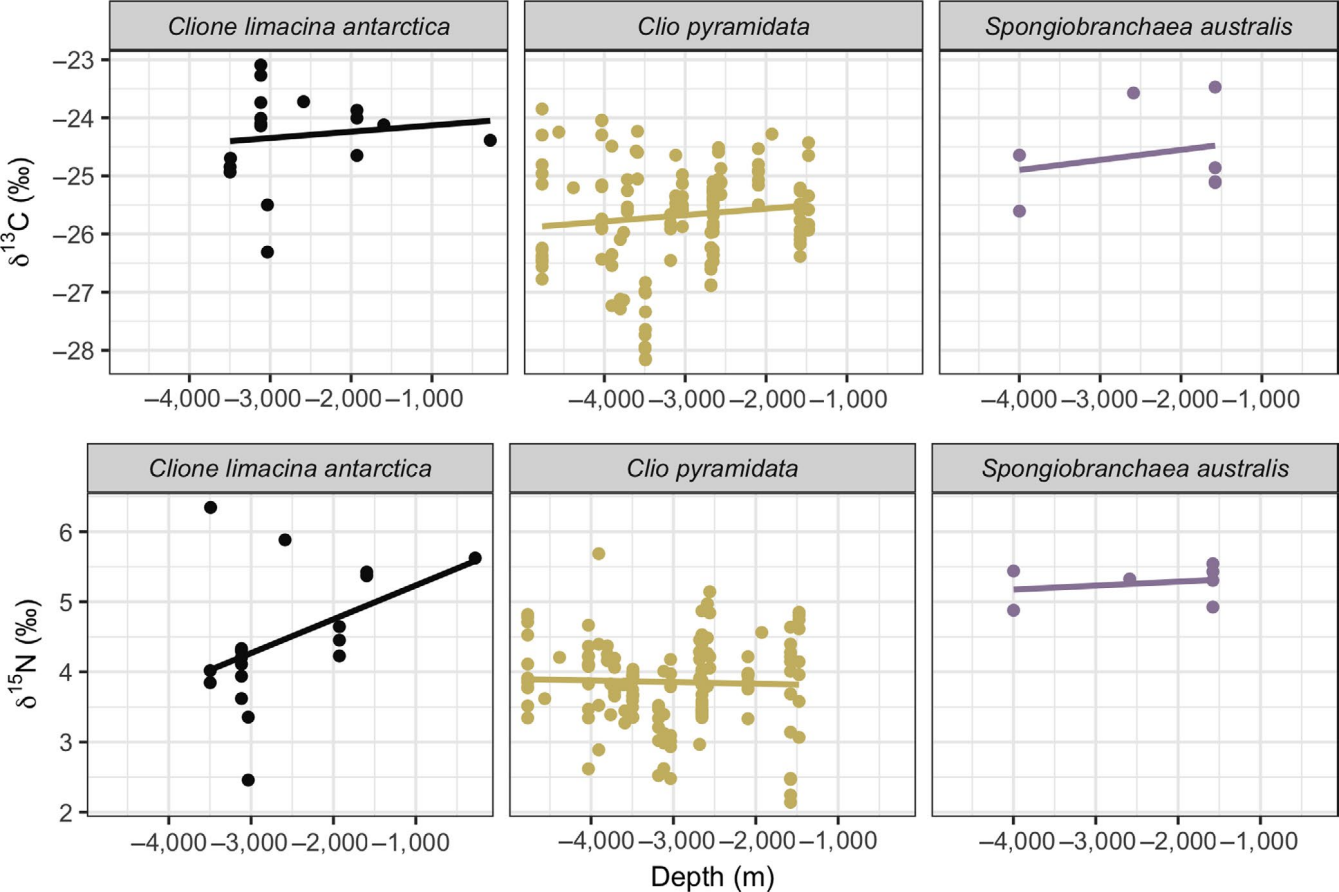
Linear relationships between δ^13^C (upper plots) and δ^15^N (lower plots) and depth (m). For *C. antarctica*: δ^13^C, *R* ^2^ = −0.05, *p* = 0.62, δ^15^N, *R* ^2^ = 0.14, *p* = 0.07; *C. pyramidata*: δ^13^C, *R* ^2^ = 0.006, *p* = 0.17, δ^15^N, *R* ^2^ = −0.006, *p* = 0.67; *S. australis*: δ^13^C, *R* ^2^ = −0.13, *p* = 0.60, δ^15^N, *R* ^2^ = −0.13, *p* = 0.59

**Figure 4:**
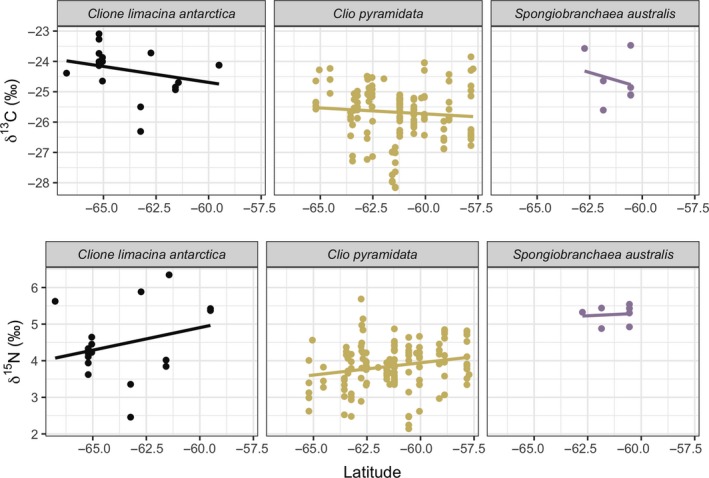
Linear relationships between δ^13^C (upper plots) and δ^15^N (lower plots) and latitude (°S). For *C. antarctica*: δ^13^C, *R* ^2^ = 0.03, *p* = 0.24, δ^15^N, *R* ^2^ = 0.02, *p* = 0.27; *C. pyramidata*: δ^13^C, *R* ^2^ = −0.0001, *p* = 0.32, δ^15^N, *R* ^2^ = 0.04, *p* < 0.05; *S. australis*: δ^13^C, *R* ^2^ = −0.14, *p* = 0.62, δ^15^N, *R* ^2^ = −0.19, *p* = 0.84

**Figure 5:**
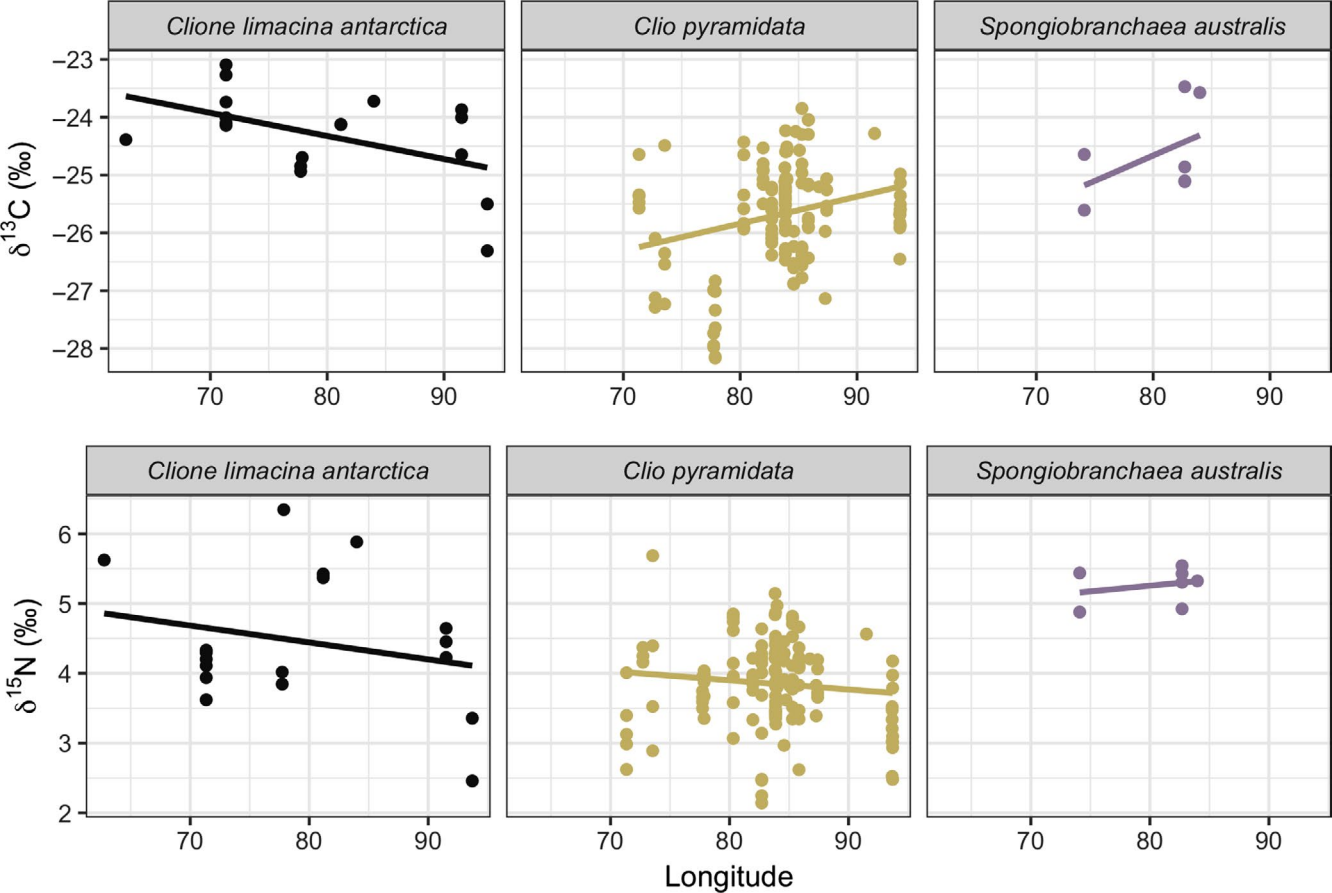
Linear relationships between δ^13^C (upper plots) and δ^15^N (lower plots) and longitude (°E). For *C. antarctica*: δ^13^C, *R* ^2^ = 0.20, *p* < 0.05, δ^15^N, *R* ^2^ = −0.001, *p* = 0.34; *C. pyramidata*: δ^13^C, *R* ^2^ = 0.06, *p* < 0.05, δ^15^N, *R* ^2^ = 0.06, *p* < 0.05; *S. australis*: δ^13^C, *R* ^2^ = 0.07, *p* = 0.28, δ^15^N, *R* ^2^ = −0.11, *p* = 0.55

δ^13^C and δ^15^N values were obtained from 185 individuals representing all three pteropod species from the RMT8 samples (*C. pyramidata*: *n* = 152; *C. antarctica*: *n* = 24; *S. australis*: *n* = 9). Pairwise comparison tests indicated significant differences among stable isotopic values associated with parameters tested (MANOVA, δ^13^C and δ^15^N values, *p* < 0.001; see Table S1). δ^13^C values were strongly associated with spatial attributes, including latitude, longitude, and depth modeled together. Variation in δ^15^N values was primarily predicted by longitude and species. Linear regression models of species‐specific relationships between each isotopic value versus spatio‐temporal variables (date, depth, latitude, and longitude; Figures 2, 3, 4, 5) showed statistically significant positive relationships for the following: *C. pyramidata* between δ^15^N and sampling dates and latitude (Figures 2 and 4, respectively), and between δ^13^C and longitude (Figure 5); and *C. antarctica* between δ^15^N and δ^13^C and sampling dates (Figure 2). Statistically significant negative relationships were measured for the following: *C. pyramidata* between δ^13^C and sampling dates and longitude (Figures 2 and 5, respectively); and *C. antarctica* between δ^13^C and longitude (Figure 5).

### 3.3 Between‐species isotopic niche widths, overlap, and body size effects

**Figure 6:**
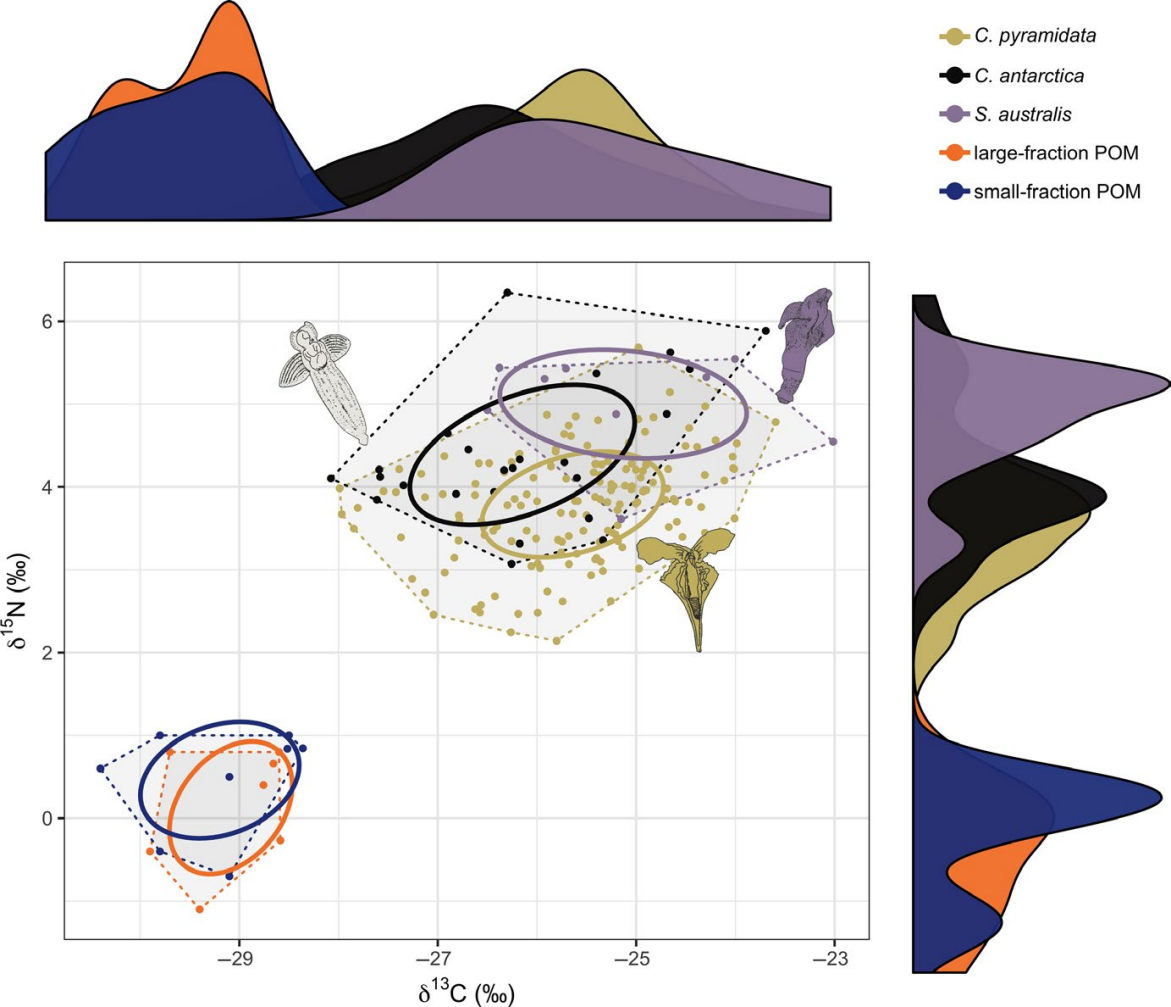
δ^13^C‐δ^15^N biplot of isotopes values, standard ellipses (solid lines; 40% probability level), convex hulls (dashed lines), and density plots (outer axes) for each pteropod species and large‐ and small‐fraction (gray) POM

Between‐species comparisons of pteropods revealed the largest mean total niche area was for *C. antarctica* (8.15‰^2^), followed by *C. pyramidata* (7.90‰^2^), and *S. australis* (4.19‰^2^) (Figure 6; see Table S2). POM exhibited mean total niche areas of 1.86‰^2^ (large fraction; *n* = 7) and 2.18‰^2^ (small fraction; *n* = 8). Mean (95% credibility limits) standard ellipse areas (SEA and SEAc) between species showed the largest estimates for gymnosomes *C. antarctica* and *S. australis* (Figure 6; see Table S2).

Probability niche overlap was high among all pteropod species (Figure 6, see Table S3), with the highest overlap observed between *C. antarctica* and *C. pyramidata* (85.6% at α = 0.95; 95.0% at α = 0.99 probabilistic niche regions (PNR)), followed by *C. antarctica* and *S. australis* (79.5% at α = 0.95; 89.9% at α = 0.99 PNR) and *C. pyramidata* and *S. australis* (48.0% at α = 0.95; 64.8% at α = 0.99 PNR). We found small niche overlap between *C. pyramidata* and large‐fraction POM (0.19% at α = 0.95; 0.65% at α = 0.99) and small‐fraction POM (0.14% at α = 0.95; 0.74% at α = 0.99).

**Figure 7:**
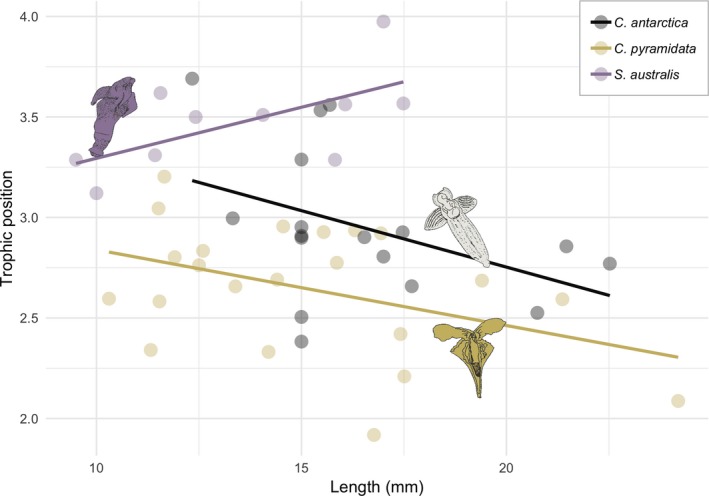
Relationship between body length (mm) and trophic position of pteropods *C. pyramidata*, *C. antarctica*, and *S. australis*. Equations for regression lines are: *C. pyramidata*, *y* = 4.74 − 0.08*x* (*n* = 21, *r* ^2^ = 0.17, *p* = 0.06); *C. antarctica*, *y* = 6.06 − 0.11*x* (*n* = 17, *r* ^2^ = 0.18, *p* = 0.09); *S. australis*, *y* = 3.88 + 0.10 (*n* = 9, *r* ^2^ = 0.39, *p* < 0.05)

Only *S. australis* revealed a positive linear relationship between body length (mm) and δ^15^N value, whereas the other two species, *C. pyramidata* and *C. antarctica*, showed negative relationships (Figure 7); only the relationship for *S. australis* was statistically significant (*r* ^2^ = 0.39, *p* = 0.05).

### 3.4 Trophic positions

**Figure 8:**
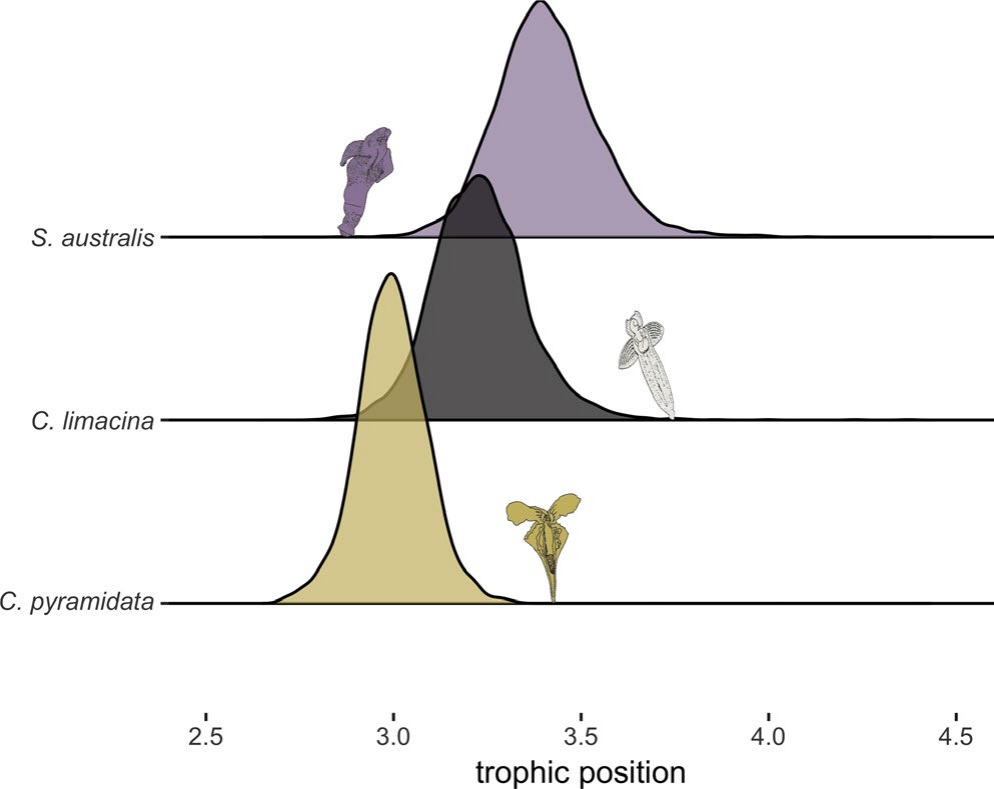
Bayesian estimates of trophic position created from 20,000 Markov Chain Monte Carlo iterations for each pteropod species

Average and modal trophic positions were determined for all species (Figure 8), with *S. australis* exhibiting the highest trophic position (TP average: 3.46 ± 0.18; TP mode = 3.39) followed by *C. antarctica* (TP average: 3.20 ± 0.17; TP mode = 3.12), and *C. pyramidata* (TP average: 2.84 ± 0.14; TP mode = 2.75).

## 4 DISCUSSION

We hypothesized that Southern Ocean pteropods would reveal variability in isotopic niche areas that reflect a priori knowledge of species‐specific feeding preferences, with specialists, the gymnosomes, exhibiting smaller niche areas than generalists, the thecosomes. We expected narrow isotopic niches for gymnosomes possessing monophagous diets, consisting of a single species or genus, with *C. antarctica* preferring to prey on *Limacina* spp. and *S. australis* preying only on *C. pyramidata*. Instead, we measured broader than expected isotopic niche areas for gymnosomes *C. antarctica* and *S. australis*, compared to the value estimated for thecosome *C. pyramidata*. The generally accepted idea that niche width positively correlates with diet breadth is based on a common assumption that has emerged from the niche variation hypothesis (Van Valen, 1965). Empirical work involving an isotopically heterogenous range of consumers has revealed broader isotopic niches occupied by populations of dietary specialists in relation to those of generalists (Flaherty & Ben‐David, 2010). Modeling efforts focused on the isotopic composition of marine assemblages have confirmed that estimations of an organism's trophic niche can be confounded by isotopic variability in prey, regardless of both the source of isotopic variation and/or the diet behavior (Cummings, Buhl, Lee, Simpson, & Holmes, 2012). Below, we discuss factors that may explain these patterns, but note that distinguishing among these mechanisms would require additional sampling and analysis and would be a fruitful direction for future work.

The isotopic variation within pteropod species measured here suggests niche partitioning and could point to their distinctive functional traits as observed through anatomical structures associated with feeding. Thecosomes such as *C. pyramidata* particle feed on marine snow by deploying an external mucus web to capture suspended materials of all sizes, which are then transported to the mouth by ciliary pathways, where particle sorting and ingestion occur (Gilmer, 1974, 1990). Gut content data from the Weddell Sea have revealed an omnivorous diet for *C. pyramidata* (Hopkins & Torres, 1989), corroborated by the broad range of δ^15^N values measured within the present study. In contrast to thecosomes, the feeding apparatus of gymnosomes varies among species. *C. antarctica* possess six buccal cones that capture prey, and a series of hooks and the radula remove and swallow the soft tissue whole from the shell (Conover & Lalli, 1972), whereas *S. australis* captures prey using two lateral arms, each possessing a series of suckers (Lalli & Gilmer, 1989). While both gymnosomes revealed broad δ^13^C value ranges, which closely matched that of *C. pyramidata*, the variable ranges in δ^15^N values displayed between each gymnosome may be a result of these species‐specific feeding strategies.

Published δ^13^C‐δ^15^N biplots featuring Southern Ocean pteropod species place *S. australis* trophically higher than any other pteropod species common to the region (Hunt et al., 2008; Table 1). By our estimates, *S. australis* was one trophic level above its prey *C. pyramidata*, however despite obtaining a similar outcome to previous isotopic work evaluating the interaction between these two species (Table 1), these results alone cannot confirm a direct trophic relationship without supplemented by other lines of evidence, such as direct observation, genetic, and/or gut content analyses (Nielsen, Clare, Hayden, Brett, & Kratina, 2018). More work is required to better understand prey size preference for this species. Meanwhile, *C. pyramidata* possessed a larger total niche area than those measured for gymnosomes, which likely reflecting omnivory.

**Table 1:** Bulk stable isotopes (δ^13^C and δ^15^N) signatures (± *SD*) averaged over all sampling sites and species, as well as results from the same Southern Ocean species from other studies

Species	Region	*n*	δ^13^C	δ^15^N	References
*C. pyramidata*	S. Kerguelen Plateau	152	−28.1 ± 0.9	3.8 ± 0.6	This study
	Lazarev Sea^a^	6	~−26 to −29.5	~1 to 2	Hunt et al. (2008)
	SW Indian Ocean, *N* of STC	3	−20.3 ± 0.5^b^	5.6 ± 0.2	Richoux and Froneman (2009)^c^
	SW Indian Ocean, mid‐STC	—	−20.7^b^	4.1	Richoux and Froneman (2009)^c^
	SW Indian Ocean, S of STC	2	−20.6^b^	0.4 ± 0.3	Richoux and Froneman (2009)^c^
*C. limacina*	S. Kerguelen Plateau	24	−28.3 ± 1.1	4.5 ± 0.9	This study
	Lazarev Sea^a^	3	~−28.5 to −29.7	~2.5 to 3.7	Hunt et al. (2008)
	E. Antarctica^d^	3	−30.8	5.1 ± 0.9	Jia et al. (2016)
*S. australis*	S. Kerguelen Plateau	9	−27.9 ± 1.0	5.3 ± 0.3	This study
	Lazarev Sea^a^	3	~−27.5 to −30	~5.5 to 6.5	Hunt et al. (2008)
	Prince Edward Islands^e^	3	—	~2.8 to 3.5^f^	Hunt et al. (2008)

Note

Some values are listed as approximate ranges where exact values and standard deviations are unavailable.

a April 2004.

b Carbonate‐corrected.

c Study pooled thecosome species which included *C. pyramidata*.

d September to November 2012.

e April 1999.

f Lipid‐corrected.

We predicted narrow isotopic niche widths for both gymnosomes relative to that of *C. pyramidata*; however, our results demonstrate significant trophic diversity not only between orders but also between gymnosome species. For *S. australis*, this may be a function of latitude, as this least‐abundant species was sampled within the narrowest latitudinal range; however, linear relationships did not reveal any statistically significant spatial correlations. We expected the isotopic niche width of both gymnosomes to be narrow because of a priori knowledge of their specialist feeding preference for thecosomes (Phleger, Nichols, & Virtue, 1997). However, the niche width of *C. antarctica* was significantly broader along the δ^15^N‐axis than hypothesized, which might be a function of their wider geographical range within our voyage transect, relative to that of *S. australis* more commonly found north of the Polar Front (PF) and the northern extent of our sampling effort (Hunt et al., 2008).

The unexpectedly wide niche area of *C. antarctica* might be a function of either diet switching to other unrecorded food sources, and/or driven by nutritional stress. One study posited that *C. limacina* diet may include other taxa when the preferred prey *Limacina* species are limited. DNA‐based approaches used to investigate prey within Arctic *C. limacina* stomach contents revealed alternative prey items, such as amphipods and calanoid copepods (Kallevik, 2013). Further, laboratory observations have demonstrated that *C. limacina* can survive between 260 and 365 days without prey due to an adaptive ability to reduce body size and metabolic rate as a consequence of efficient utilization of lipids and phospholipids stores (Böer, Graeve, & Kattner, 2006, 2007; Falk‐Petersen, Sargent, Kwasniewski, Gulliksen, & Millar, 2001). However, no studies have tested how these changes affect *C. limacina* isotopically. Among the numerous studies examining how a range of nutritional stresses affect isotopic compositions of various animals, enrichment in ^15^N is the response most likely to confound interpretations of isotopic niches, particularly given that isotopic niche metrics rely on the assumption that interindividual variability in enrichment between consumer and prey is insignificant (Karlson, Reutgard, Garbaras, & Gorokhova, 2018; McCue & Pollock, 2008). One experiment‐based study on the amphipod *Monoporeia affinis* revealed wider niche widths among specimens exposed to nutrient and chemical stress relative to control animals (Karlson et al., 2018). It is understood that increases in isotopic niche areas are a function of interindividual variability in the responses of consumers to stress exposure through changes in their metabolic pathways. Further laboratory testing is needed to understand how exposure to physiological stress, such as food limitation, can affect the magnitude of the isotopic compositions and niche metrics of *C. antarctica,* with special focus on the scale of individual. Furthermore, results of laboratory tests will need to be compared with in situ estimates of niche metrics taken from *C. limacina* sampled in years where prey species, *L. helicina*, were also abundant.

The qualitative concept of niche breadth can be fundamental to understanding the adaptive capabilities of an organism or assemblage, and is potentially useful for investigating responses of climate‐driven changes to prey availability (Sexton et al., 2017). Recently, Henschke et al., (2015) employed isotopic niche analysis of co‐occurring zooplankton and suspended POM assemblages from the Tasman Sea under three different oceanographic water types and found that omnivorous zooplankton became more carnivorous under low chlorophyll *a* conditions. They concluded that niche breadth among different zooplankton groups was a function of the responses of phytoplankton and POM to oceanographic conditions. Narrower niches have been observed among CO_2_‐enriched hydrothermal vent communities relative to controls, whereby near‐future predicted *p*CO_2_ concentrations are directly associated with decreased higher trophic species diversity and simplified habitats (Agostini et al., 2018; Vizzini et al., 2017).

*Clione antarctica* showed the highest average bulk C:N ratio (6.8 ± 1.4), which is lower than reported for spring to early summer 2012 (C:*N* = 10.2; Table 1) in East Antarctica (Jia, Swadling, Meiners, Kawaguchi, & Virtue, 2016). Seasonal build‐up of lipid deposits may be contributing to this difference, as C:N ratios in zooplankton are often positively related to lipid content (Syväranta & Rautio, 2010) which is depleted in ^13^C relative to carbohydrates and proteins (Tieszen, Boutton, Tesdahl, & Slade, 1983). Empirical evidence has shown that lipid extraction has a significant influence on stable isotope ratios of Southern Ocean pteropods, including *C. antarctica* (Weldrick et al., 2019). *Clione* spp. have developed a unique metabolic adaptation through de novo biosynthesis of large deposits of 1‐*O*‐alkyldiacylglycerol ethers (DAGE) for long‐term energy storage (Kattner, Hagen, Graeve, & Albers, 1998) necessary to cope with food limitation and overwintering survival (Böer et al., 2005; Phleger, Nelson, Mooney, & Nichols, 2001). Meanwhile, similar studies on *S. australis* (C:N = 5.1 ± 0.4, C content = 34.5%–44.2%; N content = 7.23%–8.65%) and its prey, *C. pyramidata* (C:N = 3.9 ± 0.3; C content = 24.9%–41.2%; N content = 5.4%–10.4%), have reported low to no levels of DAGE, respectively (Phleger, Nelson, Mooney, & Nichols, 1999).

Trophic positions of many aquatic species are, in many cases, more strongly predicted by body size than by taxonomy (Jennings, Pinnegar, Polunin, & Boon, 2001; Soler, Edgar, Stuart‐Smith, Smith, & Thomson, 2016) and distinctive feeding strategies specialize ontogenetically for many Southern Ocean zooplankton (Schmidt et al., 2003; Zhang et al., 2017). Generally, food preference in pteropods varies with age and season, and depends on food particle size and availability; with veliger‐ and juvenile‐aged thecosomes primarily relying on marine POM, and adults consuming a largely diatom‐based diet in spring and summer ahead of a switch to detrital materials between late autumn and winter when phytoplankton blooms are depleted (Gannefors et al., 2005; Manno, Tirelli, Accornero, & Fonda Umani, 2010). As our study focused on macrozooplankton greater than 2,000 µm, we only present adult stages for each species, and further research is required to explore size‐trophic position relationships that also incorporate earlier life stages.

For *C. pyramidata*, we did not observe an increase in trophic position with body size that would signify a possible shifting from herbivory to omnivory. Positive correlations between *C. pyramidata* abundance and phytoplankton blooms have been linked to high ingestion rates, with pteropods contributing up to 53% to total grazing impact within the Spring ice edge while contributing only 13% to total biomass in the region (Pakhomov & Froneman, 2004). Gut content analyses from Weddell Sea specimens showed *C. pyramidata* f. *sulcata* diets primarily composed of diatoms; however, larger motile organisms such as tintinnids, foraminiferans, copepods, and polychaetes appeared in larger sized *C. pyramidata*, pointing to omnivorous feeding habits (Hopkins & Torres, 1989). Fatty acid profiles linked to crustacean biomarkers have also been measured in *C. pyramidata* from the Antarctic Peninsula (Phleger et al., 2001).

For gymnosomes, our results imply a continuous change in dietary preference throughout adult stages, which may vary spatially and depend on prey availability. A laboratory‐based feeding experiment using Arctic *C. limacina* sampled in mid‐summer measured low prey assimilation rates that increased toward the end of the experiment (Boissonnot et al., 2019). Results from their study suggested that sampling period overlapped with a seasonal period of low prey availability followed by high reproductive activity and subsequent body shrinkage. Among the RMT1 samples taken from the same research program (K‐Axis) analyzed here, Matsuno, Wallis, Kawaguchi, Bestley and Swadling (submitted) identified and counted the preferred prey of *C. limacina*, *L. helicina*; however, all specimens were juveniles with an average shell diameter of 0.4 ± 0.1 mm (see Table S4), and laboratory‐based feeding experiments have shown that adult *C. antarctica* rarely consume *L. helicina* smaller than ~1 mm (Conover & Lalli, 1972). For *C. antarctica*, the negative relationship between trophic position and body size measured here could point to stress‐induced isotopic enrichment within nutrient‐limited body tissues causing body shrinkage (Boag, Neilson, & Scrimgeour, 2006). However, while this result aligns with the wider than expected niche width obtained for this species, the relationship was not statistically significant and conclusions cannot be drawn from this result.

Pteropod abundances throughout the Southern Ocean indicate highly variable yet sometimes dominant densities in proportion to other zooplankton groups, contributing up to 93% of total macrozooplankton (Hunt et al., 2008), and strongly tied to El Niño events, sea ice retreat, and the presence of prey (Thibodeau, Steinberg, Stammerjohn, & Hauri, 2019). Our maximum abundance of 272 ind. 1,000 m^−3^ for *C. pyramidata* is within the range of 2—996 ind. 1,000 m^−3^ previously recorded for other macrozooplankton samples (4.5 mm mesh size) throughout the Southern Ocean (Hunt et al., 2008). Maximum abundance for both gymnosome species combined, 47 ind. 1,000 m^−3^, was high compared to macrozooplankton samples throughout the Southern Ocean, including the maximum abundances ranging 2.0—27 ind. 1,000 m^−3^ of gymnosome species combined (Hunt et al., 2008).

Linear regressions revealed a statistically significant positive relationship between δ^13^C and longitude for *C. pyramidata,* which may relate to the cluster of enriched values overlapping the same region (just north of the sACCf on the Plateau) we measured high abundances of this species, toward the eastern flank of the Plateau. Particulate organic nitrogen (PON) measurements revealed enriched values along this region, indicating nitrate uptake which suggests new production was occurring here (Schallenberg et al., 2018). However, we also detected a statistically significant negative relationship between δ^13^C and longitude for *C. antarctica*, whose highest abundances generally overlapped with those of *C. pyramidata*. A weakly positive significant linear relationship was measured between δ^15^N and latitude for *C. pyramidata*, who displayed gradients of isotopic enrichment from deeper regions of the transect toward the coastlines and toward shallower areas toward Banzare Bank, at 60°S and 80°E. Regions along the transect where abundances were lowest (between 70°E to 80°E) overlapped with the lowest δ^13^C and δ^15^N values measured for all species, combined. Incidentally, this region, which covers the western area of the Plateau, also measured the most ^15^N‐depleted in PON, suggesting nitrogen recycling and ammonia uptake (Schallenberg et al., 2018).

The pteropod species sampled from the southern extent of the Kerguelen Plateau are among the four common species regularly found south of the PF (Loeb & Santora, 2013). The notable absence was that of adults of the most common thecosome species, *L. helicina antarctica,* and could be a result of environmental factors affecting primary productivity. A similar absence was first documented in McMurdo Sound in December 2001, a year following a 50% to 75% reduction in phytoplankton biomass and high sea ice cover which resulted in nutrient stress‐related decreases in metabolic rates in both *L. helicina antarctica* and its monophagous predator *C. antarctica* (Seibel & Dierssen, 2003). In 1989, low densities were also recorded in the East Antarctic and may be attributed to low chlorophyll *a* biomass resulting from late winter sea‐ice retreat (Hunt et al., 2008). Satellite observations for the region and period surveyed here exhibited comparatively low average chlorophyll *a* concentration, ranging from 0.45 to 0.55 mg/m^3^ (see Figure S1 for monthly interannual time series (2008–2017) of average chlorophyll *a* concentration from satellite observations). However, in 2015, the Kerguelen Plateau region experienced lower than average maximum chlorophyll *a* concentration (<0.5 mg/m^3^) preceded by much lower averages (<0.3 mg/m^3^) since December 2012. These values are less than the lowest value of 1.0 mg/m^3^ observed by Seibel and Dierssen (2003) the year prior to their observed absence of *L. helicina*. It is possible that with chlorophyll *a* concentration decreasing to less than 0.8 mg/m^3^ since 2008, this region may not provide enough resources to sustain growth and development of *L. helicina* assemblages beyond juvenile stage.

## 5 CONCLUDING REMARKS

While the Southern Ocean supports a range of diverse ecosystems, it is also among the most rapidly changing regions on a global scale, and it is an ongoing challenge to predict how these ecosystems and species will respond (Murphy et al., 2012). Climate‐driven changes in ocean temperature, ocean frontal positions, seasonal ice cover, and CO_2_ uptake are expected to significantly alter abundance, distribution, and trophodynamics of key polar organisms (Constable et al., 2014), including pteropods, as a response to changing metabolic fitness, prey availability and diversity, and competition. Given the complex spatial and temporal dynamics of the Southern Ocean, future research focused on understanding species responses due to human‐induced physical and chemical changes need to be small scale and regionally comparative (Allan et al., 2013). Monitoring variation between pteropod species in their isotopic niches coupled with their regional abundance and distribution could play a key role in understanding oceanographic and ecological drivers of change, for instance, due to their intraspecific reliance on sea ice primary production (Jia et al., 2016), and close associations with ocean frontal zones and biogeochemical provinces (Burridge et al., 2017). Additionally, such work will need to account for the magnitude of phenotypic plasticity in pteropods, including if and how their metabolic responses to multiple stressors of climate change are driving variability in isotopic niches. The work presented here concludes that isotopic niches in Southern Ocean pteropod communities are not merely driven by diet diversity nor follow the niche variation hypothesis of Van Valen (1965). To our knowledge, the present study represents the first comprehensive assessment involving measurement and interpretation of isotopic niche widths among pteropod assemblages. Our findings highlight the utility of employing isotopic niche metrics and dispersion analyses to reconstruct the trophic structure of co‐occurring Southern Ocean pteropods, and the need to expand this research to understand drivers of diet behavior.

## CONFLICT OF INTEREST

None declared.

## AUTHOR CONTRIBUTIONS

CKW conceived ideas, designed methodology, gathered pteropod observations, conducted analyses, led the writing of the manuscript; RT and KMS contributed critically to all draft versions; DMD provided POM observations. All authors gave final approval for publication.

## Data Availability

All isotopic signatures will be made available at Australian Antarctic Division Data Centre, https://data.aad.gov.au/aadc/; R scripts can be accessed at https://github.com/ChevaldeMer/Weldrick_etal_Ecology_and_Evolution_2019.
