# Supplementary material for: Trophodynamics of Southern Ocean pteropods on the southern Kerguelen Plateau
Source: Ecol Evol. 2019 Jun 20;9(14):8119–32. doi: 10.1002/ece3.5380 (PMC6662407; doi:10.1002/ece3.5380)
Supplement: Supplementary file 1 [file ECE3-9-8119-s001.docx]

**Supplementary materials**

Table S1 Summary of multivariate analysis of variance (MANOVA) results for isotopic variations among bulk samples.

| Source of variation | δ^13^C (‰) | | | |  | δ^15^N (‰) | | | |
| --- | --- | --- | --- | --- | --- | --- | --- | --- | --- |
|  | *df* | SS | MS | *F* |  | *df* | SS | MS | *F* |
| Latitude | 1 | 0.39 | 0.39 | 0.71*^NS^* |  | 1 | 0.90 | 0.90 | 2.71*^NS^* |
| Longitude | 1 | 2.44 | 2.44 | 4.42* |  | 1 | 4.51 | 4.51 | 13.50*** |
| Depth | 1 | 2.06 | 2.06 | 3.72*^NS^* |  | 1 | 1.32 | 1.32 | 3.94* |
| Species | 2 | 1.36 | 0.68 | 1.23*^NS^* |  | 2 | 18.62 | 9.31 | 27.84*** |
| Latitude x longitude | 1 | 16.17 | 16.17 | 29.25*** |  | 1 | 0.15 | 0.15 | 0.44*^NS^* |
| Latitude x depth | 1 | 0.001 | 0.001 | 0.002*^NS^* |  | 1 | 2.13 | 2.13 | 6.37* |
| Longitude x depth | 1 | 0.05 | 0.05 | 0.09*^NS^* |  | 1 | 0.46 | 0.46 | 1.37*^NS^* |
| Latitude x species | 2 | 5.58 | 2.80 | 5.05** |  | 2 | 0.89 | 0.44 | 1.33*^NS^* |
| Longitude x species | 2 | 0.39 | 0.20 | 0.35*^NS^* |  | 2 | 0.16 | 0.08 | 0.24*^NS^* |
| Depth x species | 1 | 1.26 | 1.26 | 2.28*^NS^* |  | 1 | 1.18 | 1.18 | 3.54*^NS^* |
| Latitude x longitude x depth | 1 | 18.49 | 18.49 | 33.46*** |  | 1 | 0.01 | 0.01 | 0.01*^NS^* |
| Latitude x longitude x species | 1 | 0.99 | 0.99 | 1.79*^NS^* |  | 1 | 0.02 | 0.02 | 0.06*^NS^* |
| Latitude x depth x species | 1 | 0.99 | 0.99 | 1.80*^NS^* |  | 1 | 1.29 | 1.29 | 3.87*^NS^* |
| Longitude x depth x species | 1 | 2.66 | 2.66 | 4.80* |  | 1 | 2.72 | 2.72 | 8.14** |
| Full model | 1 | 0.15 | 0.15 | 0.27*^NS^* |  | 1 | 2.57 | 2.57 | 7.68** |
| Residuals | 146 | 80.69 | 0.55 |  |  | 146 | 48.80 | 0.33 |  |

﻿* *p* < 0.05; ** *p* < 0.01; *** *p* < 0.001; *^NS^* no significant difference

Table S2 Total area (TA), standard ellipse area (SEA), and SEA corrected for small sample size (SEAc) for each pteropod species and large- and small-fraction POM.

|  | *C. pyramidata* | *C. antarctica* | *S. australis* | POM (large) | POM (small) |
| --- | --- | --- | --- | --- | --- |
| TA (‰^2^) | 7.90 | 8.15 | 4.19 | 1.86 | 2.18 |
| SEA (‰^2^) | 1.66 | 2.55 | 2.22 | 1.22 | 1.47 |
| SEAc (‰^2^) | 1.67 | 2.67 | 2.53 | 1.46 | 1.71 |

Table S3 Niche overlap metric estimates (%) of 10,000 Monte Carlo sampling draws from the prior parameters. Values represent calculations made at 95 and 99 % probabilistic niche region (PNR; α = 0.95, 0.99).

|  |  | *C. pyramidata* | *C. antarctica* | *S. australis* | POM (large) | POM (small) |
| --- | --- | --- | --- | --- | --- | --- |
| PNR: 95% | *C. pyramidata* | -- | 85.60 | 47.95 | 0.19 | 0.14 |
|  | *C. antarctica* | 68.35 | -- | 62.44 | 0.05 | 0.06 |
|  | *S. australis* | 49.68 | 79.47 | -- | 0.02 | 0.00 |
|  | POM (large) | 0.13 | 0.35 | 0.03 | -- | 82.68 |
|  | POM (small) | 0.12 | 0.32 | 0.00 | 74.03 | -- |
| PNR: 99% | *C. pyramidata* | -- | 95.00 | 64.81 | 0.65 | 0.74 |
|  | *C. antarctica* | 83.87 | -- | 75.02 | 0.79 | 0.86 |
|  | *S. australis* | 71.04 | 89.93 | -- | 0.05 | 0.03 |
|  | POM (large) | 0.36 | 1.37 | 0.05 | -- | 90.92 |
|  | POM (small) | 0.40 | 1.73 | 0.01 | 85.00 | -- |

Figure S1 Interannual monthly time series of average chlorophyll *a* concentration (mg m^-3^) for December, January and February (2008-2017), derived from MODIS-Aqua satellite data distributed by NASA (Level 3 binned, 4 km resolution; https://oceandata.sci.gsfc.nasa.gov/MODIS-Aqua/L3BIN/).

*Details of biomass estimations conducted on both RMT1+ 8 pteropods sampled from K-axis voyage*

Carbon biomass estimates (mg C m^-3^) were calculated as a product of abundances (converted to ind. m^-3^) estimated by Matsuno et al. (in prep) and this study (using RMT1+8 pteropod samples, respectively) and dry weights (DW, in mg) for *L. helicina* and *L. retroversa*, and wet weights (WW, in mg) for *C. pyramidata*, *C. limacina*, and *S. australis*, from pre-determined length-to-weight equations derived from general shell shapes and body lengths (in mm) of major pteropod groups (Bednaršek, Mozina, Vogt, O’Brien, & Tarling, 2012). Conversion factors of 0.28 and 0.25 were multiplied to convert WW to DW and DW to carbon, respectively (Davis and Wiebe, 1985; Larson, 1986).

The total biomass estimated for all five species sampled was 1.89 mg C m^-3^ (Table 1), which was mostly comprised by *C. pyramidata* (94.1%), followed by juvenile aged *L. helicina* (5.6%), *C. antarctica* (0.27%), *S. australis* (0.02%), and *L. retroversa* (0.001%).

Table S4. Details of sampling net, mean length (body or shell), length-to-weight equation used (described in Bednaršek et al. 2012), total abundance, and carbon biomass estimates for each pteropod species.

| Species | RMT | Mean length (mm±SD) | Equation  (DW or WW) | Abundance (ind. m^-3^) | Biomass  (mg C m^-3^) | Study |
| --- | --- | --- | --- | --- | --- | --- |
| *C. pyramidata* | 8 | 15.5 (4.3) | $WW=0.2152\cdot L^{2.293}$ | 0.06 | 1.78 | This study |
| *C. antarctica* | 8 | 17.8 (3.6) | $WW={10}^{(2.533\cdot\log\left( L \right)-3.89095)\cdot{10}^{3}}$ | 0.003 | 0.005 | This study |
| *L. helicina* (juvenile) | 1 | 0.44 (0.06) | $DW=0.137\cdot D^{1.5005}$ | 2.69 | 0.107 | Matsuno et al. in prep |
| *L. retroversa* | 1 | 1.51 (0.04) | $WW=0.000194\cdot L^{2.5473}$ | 0.14 | 2.10e-05 | Matsuno et al. in prep |
| *S. australis* | 8 | 14.1 (2.4) | $WW={10}^{(2.533\cdot\log\left( L \right)-3.89095)\cdot{10}^{3}}$ | 0.0002 | 0.0004 | This study |
